# Role of Strain-Induced Microscale Compositional Pulling on Optical Properties of High Al Content AlGaN Quantum Wells for Deep-Ultraviolet LED

**DOI:** 10.1186/s11671-022-03652-0

**Published:** 2022-01-15

**Authors:** Shiqiang Lu, Zongyan Luo, Jinchai Li, Wei Lin, Hangyang Chen, Dayi Liu, Duanjun Cai, Kai Huang, Na Gao, Yinghui Zhou, Shuping Li, Junyong Kang

**Affiliations:** Fujian Key Laboratory of Semiconductor Materials and Applications, CI Center for OSED, College of Physical Science and Technology, Xiamen University, Xiamen, 361005 China

**Keywords:** AlGaN, DUV, MQWs, Strain, Compositional pulling

## Abstract

A systematic study was carried out for strain-induced microscale compositional pulling effect on the structural and optical properties of high Al content AlGaN multiple quantum wells (MQWs). Investigations reveal that a large tensile strain is introduced during the epitaxial growth of AlGaN MQWs, due to the grain boundary formation, coalescence and growth. The presence of this tensile strain results in the microscale inhomogeneous compositional pulling and Ga segregation, which is further confirmed by the lower formation enthalpy of Ga atom than Al atom on AlGaN slab using first principle simulations. The strain-induced microscale compositional pulling leads to an asymmetrical feature of emission spectra and local variation in emission energy of AlGaN MQWs. Because of a stronger three-dimensional carrier localization, the area of Ga segregation shows a higher emission efficiency compared with the intrinsic area of MQWs, which is benefit for fabricating efficient AlGaN-based deep-ultraviolet light-emitting diode.

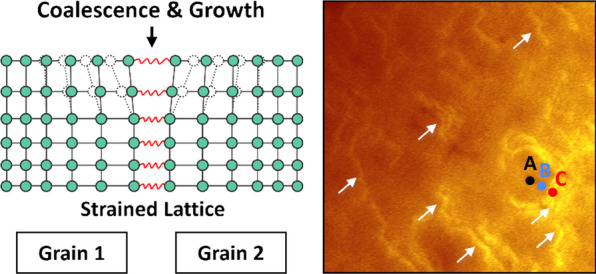

## Introduction

AlGaN-based deep-ultraviolet (DUV) light-emitting-diodes (LEDs) have various applications including sterilization and disinfection, water and air purification, medical diagnostics, high density optical recording, information sensing, bio-chemistry, and security [1–3]. Especially after the outbreak of COVID-19 pandemic, DUV LEDs have rapidly received expanding academic and industrial interests in the field of global public health [4, 5]. Moreover, according to the International Minamata Convention on Mercury, most of traditional DUV light sources that contain toxic mercury were prohibited in 2020 [6]. Therefore, the solid-state AlGaN-based DUV LEDs with high efficiency and low cost are highly and immediately desired.

Numerous studies have focused on improving the performance of AlGaN-based DUV LEDs in the past decades [1, 2, 7–10]. However, at present, the efficiency and power of AlGaN-based DUV LEDs are still relatively low compared with their visible counterparts that constructed by InGaN and GaN. Most of the reported external quantum efficiency (EQE) of AlGaN-based DUV LEDs is below 20% [11, 12]. Another problem about the AlGaN-based DUV LEDs is the commonly observed multiple or asymmetrical emission, even in the high-performance devices [11, 13–15]. Similar phenomenons are generally observed in the InGaN alloy system and have been widely ascribed to the local Indium cluster induced by compositional segregation [16–19]. However, few studies concern the mechanism contributing to the multiple or asymmetrical spectra of AlGaN DUV materials. It is known that large strain would be introduced into the AlGaN materials and devices during the epitaxial growth and cooling down processes due to the large lattice and thermal mismatch between AlGaN and sapphire substrate [20]. The existence of large strain further influences the incorporation of Al and Ga atom into the lattice, which is known as compositional pulling effect. The compositional pulling effect was commonly observed in the InGaAsP and InGaN alloy system [21, 22]. Previous studies find a bilayer nature of AlGaN film grown on the sapphire due to the compositional pulling effect, in which a compositional transition region and a compositional uniform region were observed [23, 24]. However, most of these researches about compositional pulling are focus on the AlGaN epilayer or heterojunction [25]. Far less attention has been paid to the influence on the properties of the multiple quantum wells (MQWs), which is more critical for the fabrication of DUV LEDs and commercial applications.

In this paper, we conduct systematic study for the strain-induced microscale compositional pulling effect on the structural and optical properties of high Al content AlGaN MQWs combining characterization and simulation. Real-time monitoring curve of metal organic vapor phase epitaxy (MOVPE) was analyzed to determine the growth process of MQWs. Atomic force microscope (AFM), X-ray diffraction (XRD) and transmission electron microscope (TEM) were performed to characterize the structure and crystalline quality of MQWs. Microscopic Cathodoluminescence (CL) and Raman spectra were used to investigate the strain-induced microscale compositional pulling effect and corresponding optical properties. To explore the influence of strain in the epitaxy and Ga segregation of AlGaN, first principle simulations based on the frame work of density-functional theory (DFT) were also conducted.

## Experimental and Simulation Methods

**Fig. 1:**
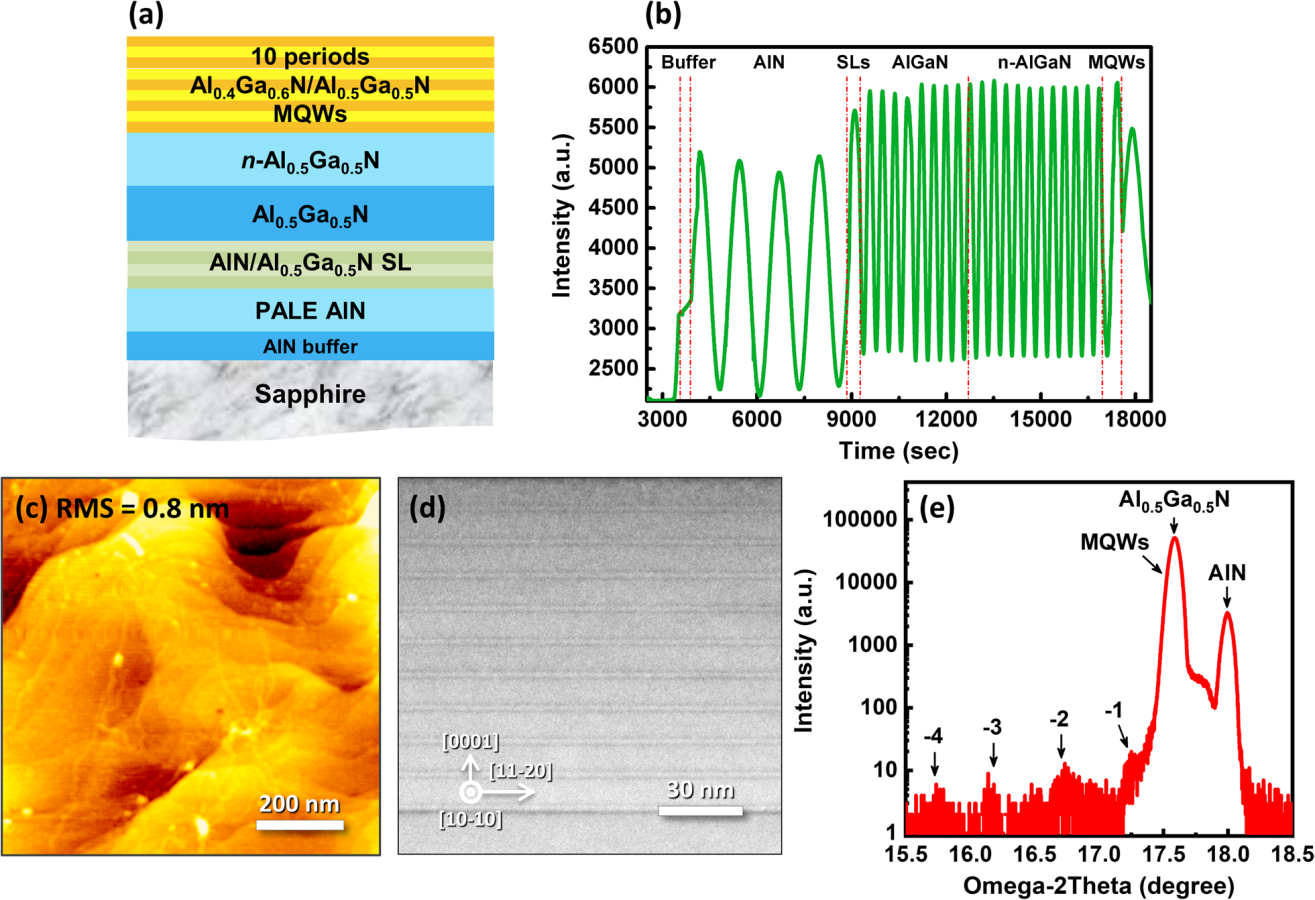
**a** Schematic of Al_0.4_Ga_0.6_N/Al_0.5_Ga_0.5_N MQWs epitaxial structure. **b** Real-time monitoring curve of the complete growth process for the sample according to MOVPE. **c** SEM image, **d** AFM surface morphology, **e** cross-sectional TEM image, and **f** HRXRD (0002) *ω*/2*θ* scan of the AlGaN MQWs

The high Al content AlGaN MQWs was grown on *c*-plane sapphire substrate via MOVPE in a vertical Thomas Swan system (3 × 2 inch CCS Aixtron). The source precursors were trimethylaluminum (TMA), trimethylgallium (TMG) and ammonia (NH_3_). Silane (SiH_4_) was used as n-type dopant source and hydrogen (H_2_) as the carrier gas. Figure 1a shows the schematic diagram of the sample structure. First, a thin AlN buffer layer was deposited on sapphire as the nucleation layer. Subsequently, a high-quality AlN layer was grown by the pulsed atomic layer epitaxy method (PALE), followed by AlN/Al_0.5_Ga_0.5_N superlattice (SL) layer, undoped Al_0.5_Ga_0.5_N layer and Si-doped n-type Al_0.5_Ga_0.5_N layer. Finally, 10 periods Al_0.4_Ga_0.6_N/Al_0.5_Ga_0.5_N MQWs were grown.

The structure and crystalline quality of MQWs were characterized by a high-resolution X-ray diffractometer (HRXRD, PANalytical X’pert PRO MRD Holland) with an X-ray wavelength of 0.154056 nm using Cu K*α* radiation, and a scanning/transmission electron microscope system (Thermo Scientific Talos F200X S/TEM). The surface morphology of AlGaN MQWs was characterized by SEM using Carl Zeiss FE-SEM SIGMA HD system, and AFM using the Seiko SPA400 system. CL spectra and images were obtained by using an electron gun (Orsay Physics “Eclipse” FEB Column) to excite the MQWs and the emitted light was dispersed by a 320 mm focal-length monochromator (Horiba Jobin Yvon iHR320) through an optical fiber. Raman spectra were collected by a Raman microscope (WITec alpha 300RA) with a 488 nm laser.

To explore the influence of strain in the epitaxy of AlGaN, the first principle simulations were carried out using the Vienna ab-initio simulation package (VASP) in the frame work of DFT [26, 27]. The Perdew–Burke–Ernzerhof (PBE) generalized gradient approximation (GGA) was used for the exchange–correlation interactions among the electrons [28]. Ga-3d electrons were treated as part of valence electrons. A 6 × 6 × 2 Monkhorst–Pack grid of *k* points was used for sampling the Brillouin zone, and a cutoff energy of 520 eV was used to expand the electronic wavefunctions, which was sufficient for the plane wave basis to achieve energy convergence results. The geometry optimizations were performed by using the conjugate gradient algorithm with convergence energy of 1 × 10^–3^ eV and 1 × 10^–4^ eV for ions and electrons, respectively. An Al_0.5_Ga_0.5_N slab model generated by 4a × 4b × 3c primitive cells was constructed for the simulation. A vacuum layer about 25 Å was applied, which was determined to be sufficiently large to avoid interaction between neighboring supercells. For the un-strained slab model, the lattice parameters and atomic coordinates were based on the bulk structure, which was optimized by relaxing all the degrees of freedom. And then, the tensile strain was applied based on the un-strained slab model. At the step of adsorption simulation, the Al_0.5_Ga_0.5_N under layer was fixed and the additional adsorbents were allowed to relax to minimize the total energy of the system.

## Results and Discussion

To determine the growth process of sample, real-time monitoring curve recorded during the MOVPE growth was first discussed, as shown in Fig. 1b. In the initial step, an approximately 20 nm buffer layer was grown for nucleation and the reflected signal was enhanced gradually due to the higher refractive index of AlN than the sapphire substrate. By introducing the PALE method, a remarkable increased amplitude appeared after the growth of buffer layer, which indicates the ending of the coalescence of the initial nucleating islands and the growth of high quality AlN layer with a gradually smoothed surface. Subsequently, the intensity of interference oscillation further increased during the growth of SLs and undoped AlGaN. After that, the oscillation intensity is maintained steady and uniform until the end of the growth process for n-AlGaN and MQWs. This monitoring curve indicates that our epitaxial strategy leads to a two-dimensional layer-by-layer growth and a smooth surface of sample, which can be further confirmed by the AFM results. As shown in Fig. 1c, well-defined steps and terraces can be observed on the smooth surface, suggesting that the step-flow growth mode has occurred. The root-mean-square (RMS) roughness value of the surface is just 0.8 nm, demonstrating the atomically flat surface and good crystal quality of MQWs.

Figure 1d illustrates the cross-sectional HRTEM image of the MQWs structure. Evidently, the abrupt interfaces and good periodicity between the well and the barrier layers can be clearly observed. The width of well and barrier is about 3.0 nm and 10.1 nm, respectively, which agree well with the growth parameters and the design expectation. A series of satellite peaks until fourth order can be resolved from the HRXRD (0002) *ω*/2*θ* scan curve, as shown in Fig. 1e, further demonstrating the formation of sharp interface and good periodicity of MQWs. By analyzing the satellite peaks via Vegard’s law [29], the period thickness of MQWs was further determined to be 12.1 nm, which is similar to that obtained from the analyses of HRTEM. Because only 10 periods of quantum wells were grown, the intensity of satellite peaks is relatively low. The 0th satellite peak cannot be resolved from the diffraction peak of n-type Al_0.5_Ga_0.5_N layer due to the close Al composition.

**Fig. 2:**
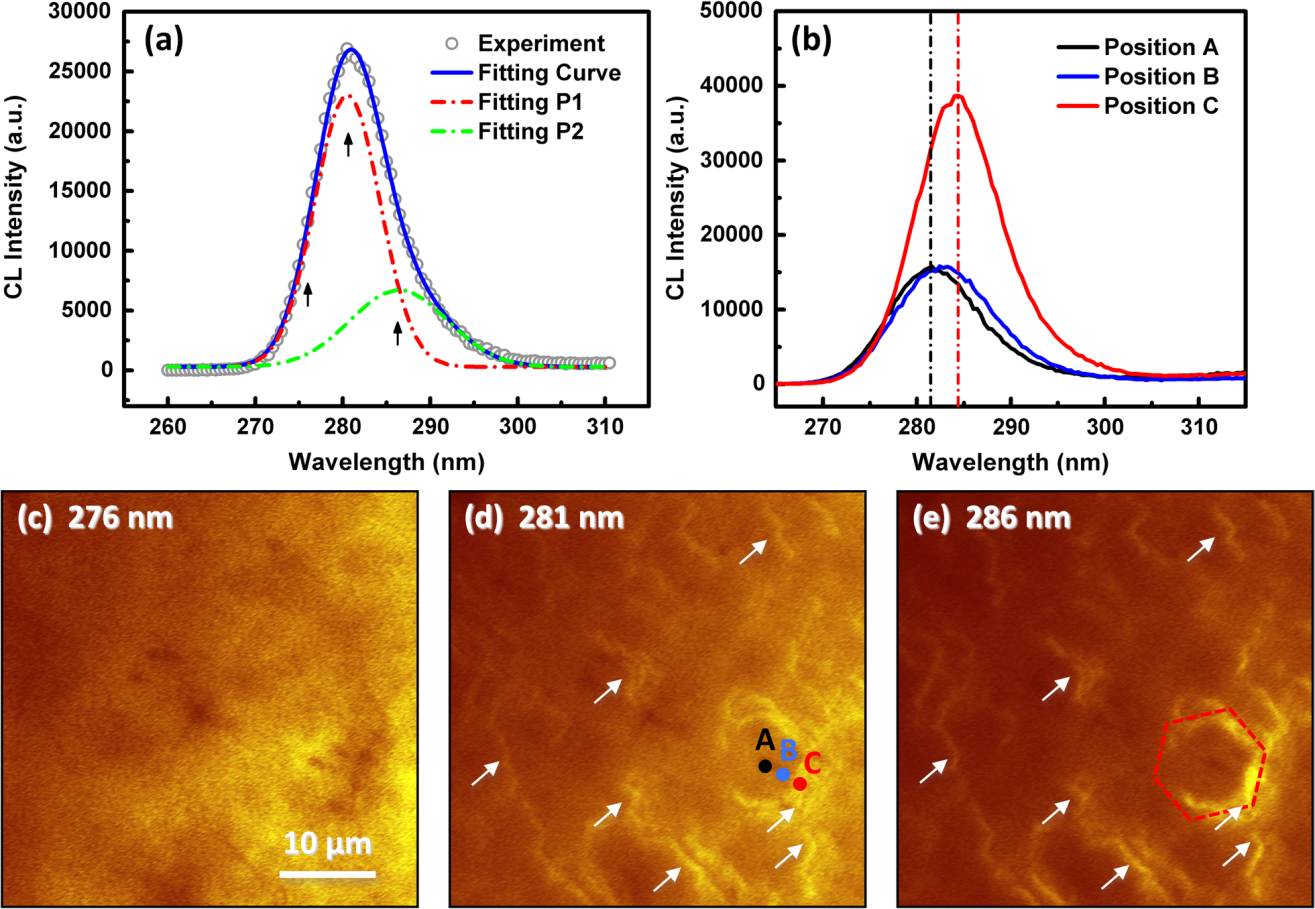
**a** Experimental and Gaussian-fitted CL spectra of MQWs. Black arrows indicate the wavelength used for the mapping. **b** CL spectra collected in Position A, B and C, and monochromatic CL mapping images taken in the same area at 285 K using the wavelengths of **c** 276 nm, **d** 281 nm and **e** 286 nm

Figure 2a shows the room temperature CL spectrum of MQWs. Evidently, the spectrum shows an intense CL emission near 281 nm, with an obvious asymmetrical feature. One dominant peak located at 280.5 nm (4.432 eV, namely P1) and a shoulder peak centered at 286.4 nm (4.341 eV, namely P2) were identified by fitting the CL spectrum with Gaussian function, as shown by the dash-dot lines in Fig. 2a. To figure out the origin of these two emission peaks, spatially resolved monochromatic CL images were conducted at 276 nm, 281 nm, and 286 nm, respectively (as black arrows indicate). Based on the monochromatic CL mapping images (Fig. 2c–e), one can find that the higher energy emission around 276 nm, which is mainly composed of P1, is uniformly come from the whole area of MQWs. However, for the lower energy emission around 281 nm, which is contributed by both P1 and P2, the emission intensity shows an inhomogeneous microscale distribution. As indicated by the white arrows, some areas with boundary-like feature show much more intense emission. And the most intense emission comes from the edge of a hexagonal area, as illustrated by the red dash line. With further increasing the wavelength of CL mapping to 286 nm, the P1 component is reduced and P2 component becomes dominant. Hence, the intensity around the boundary-like area and the edge of hexagonal area is increased ulteriorly. To further understand this inhomogeneous microscale distribution, we collected the single CL spectrum in three different positions in Fig. 2d (Position A, B, and C), and the spectra are shown in Fig. 2b. From the center (Position A), to the edge (Position C) of the hexagonal area, the emission peaks redshift about 2.9 nm (45 meV) from 281.5 nm (4.416 eV) to 284.4 nm (4.371 eV), combining with an intensity enhancement about 2.22 times. These observations demonstrate that the asymmetrical spectrum comes from two different types of emission. P1 comes from the intrinsic emission of MQWs, and P2 may have different origin.

**Fig. 3:**
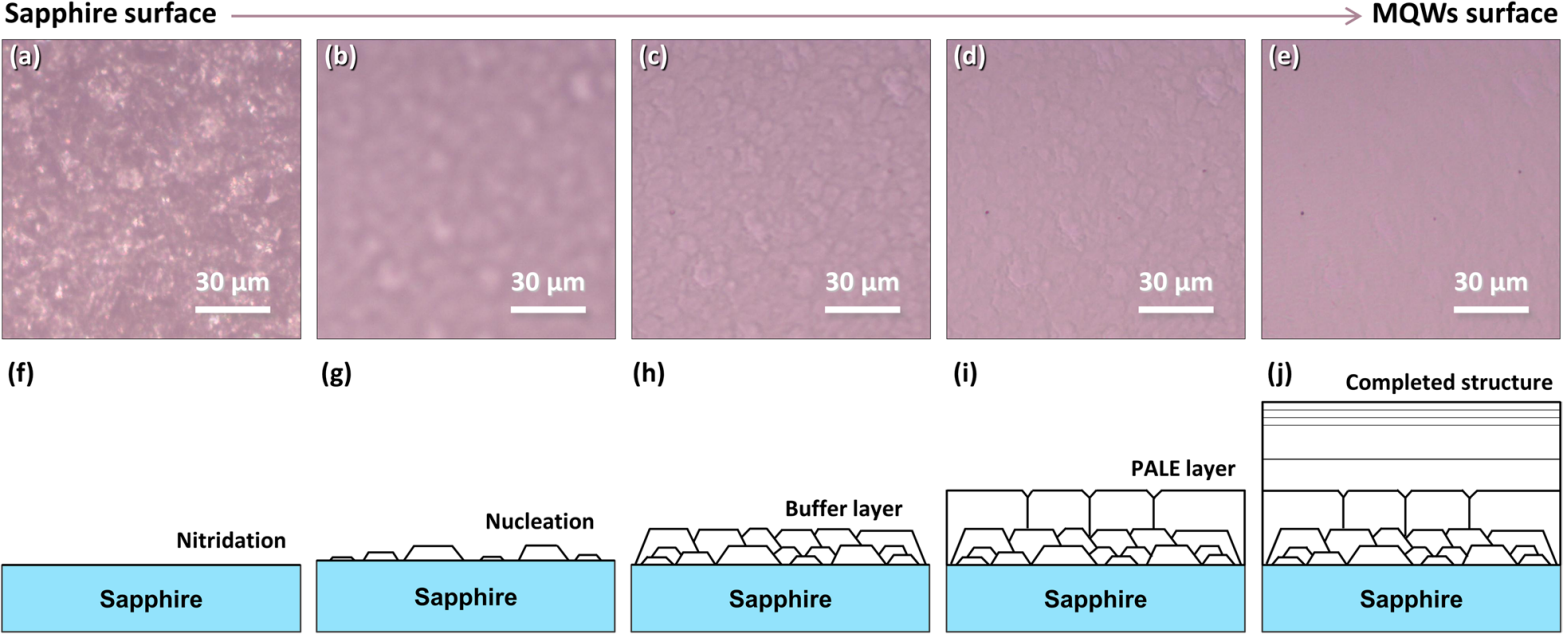
**a**–**e** Optical micrographs of the MQWs sample with the focus points changed from the sapphire surface to the MQWs surface. One can observe that the grain size is similar with the emission pattern in the monochromatic CL mapping images. **f**–**j** The schematic of two-step epitaxial growth process for nitride material

Considering epitaxial growth mechanism of group-III nitride materials, we speculate that the origin of P2 may be related to the structural evolution in the growth process. As we known, due to the large lattice and thermal mismatch between group-III nitride and sapphire substrate, it is difficult to grow nitride material directly on the sapphire. A special two-step growth technology is commonly used to grow high-quality nitride material with smooth surface, as indicated in Fig. 3a–j. Firstly, a low temperature buffer layer is deposited on the sapphire for the nucleation, then the temperature is increased above 1000 °C for the further nucleation and crystallization, followed by the coalescence of nucleation islands through lateral growth. Finally, the growth mode changes from three-dimensional to quasi-two-dimensional growth [30, 31]. By gradually changing the focus positions from the sapphire layer to the MQWs layer, the typical optical micrographs representing two-step epitaxial processes were collected (Fig. 3a–e). From these optical micrographs, one can clearly observe the coalescence of islands and grains, as well as the growth-mode transformation. A lot of mosaic grains were introduced into the material during growth, which could be the origin of P2 emission because they share the similar boundary-like or hexagonal-shape-like features. Moreover, one also can observe that the grain size (about 10 μm) and density in the opical micrographs are similar with the emission patterns in the monochromatic CL mapping images, which further confirm the fundamental origin of P2 emission.

Similar multiple or asymmetrical spectra and inhomogeneous emission distributions were commonly observed in the InGaN alloy system. This phenomenon has been mainly ascribed to the local Indium cluster induced by compositional segregation [16–19], or the compositional pulling effect caused by mismatch strain [22, 32]. For AlGaN alloy system, the Al composition has been found to segregate around dislocation lines between the crystal grain boundaries due to the non-conforming orientation of crystal columns including tilt and twist [33–35]. In our AlGaN-based MQWs sample, the longer emission wavelength of P2 suggests that it may be originated in the segregation with higher Ga composition instead of with higher Al composition. Moreover, the dislocations in AlGaN alloy are basically nonradiative [36, 37]. Again, this is not consistent with our CL observation, which shows an obvious enhancement of P2 emission. Based on the above analysis, one can exclude the dislocation-induced segregation for our AlGaN MQWs.

**Fig. 4:**
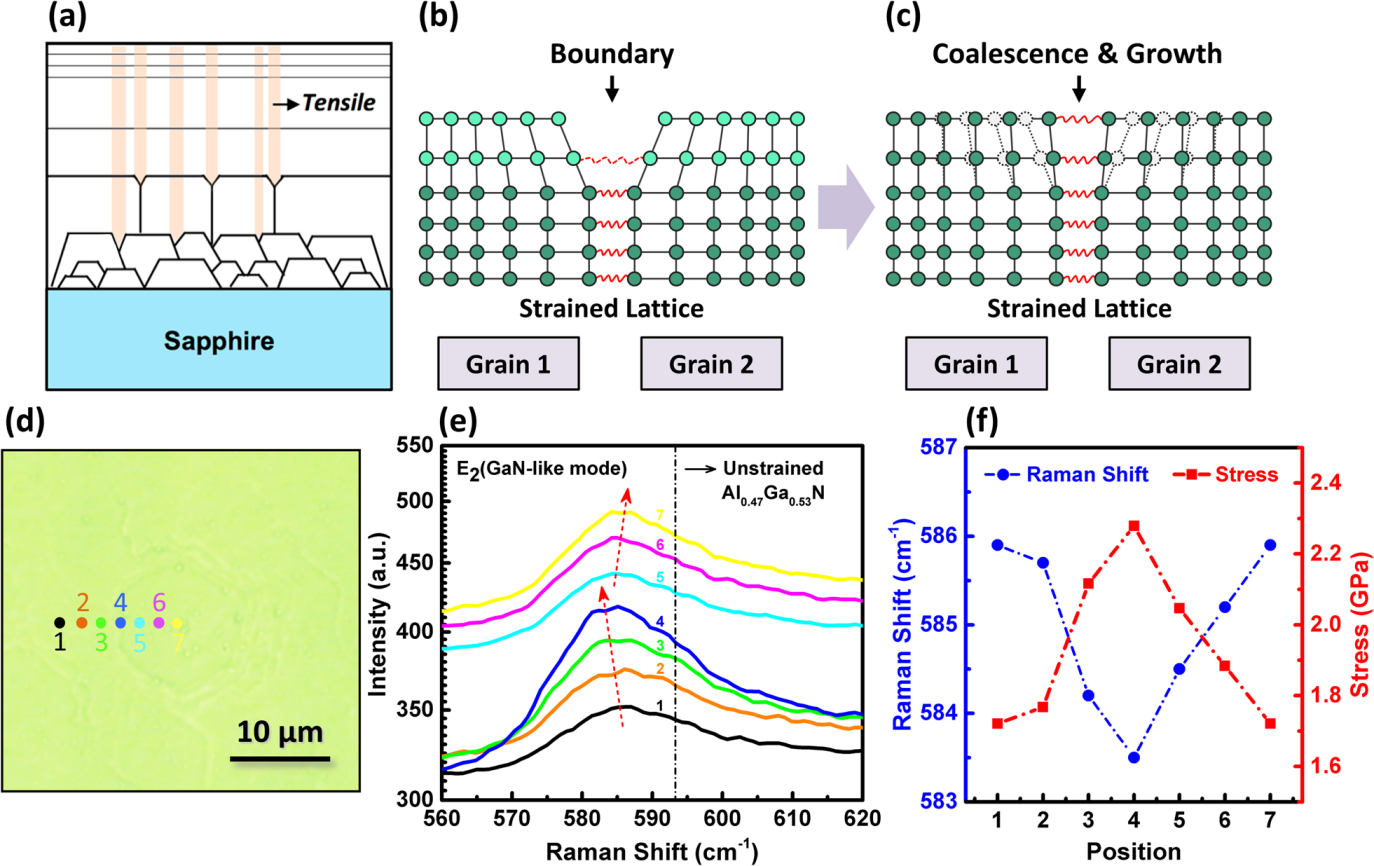
**a** Schematic of the tensile strain field in the epitaxial layer. **b**, **c** Mechanism of the tensile strain generation and extension with the grain boundary formation and growth. **d** Optical image of the hexagonal area of MQWs. The seven points indicate the positions for taking Raman spectra. **e** Raman spectra, **f** Raman shift of *E*_2_ (GaN-like) mode and calculated stress of the seven positions

Recently, some researches have demonstrated that during the growth of AlGaN layer, strong compressive strain can pull Ga atom from the AlGaN epilayer to reduce the strain energy, leading to the growth of AlGaN alloy with high Al content [23, 38]. As an analogy, if there exists a strong tensile strain field, Al atom may also be pulled out from AlGaN, resulting in a higher Ga composition. As illustrated in Fig. 4a–c, a lot of grain boundaries are produced in the epitaxial layer due to the three-dimensional growth mode in the initial stage. During the grain boundary formation and coalescence, the step edges of two adjacent grains get closer with each other, and a short-range attractive interaction between two step edges becomes strong enough to build up a tensile strain. With the continuous growth of grains, the quasi-two-dimensional growth mode is established and the tensile strain field also reaches a steady-state value, finally extending through the epitaxial layer [39, 40]. Therefore, based on these understandings of strain generation mechanism, one can draw a conclusion that the observed local variation in the emission energy and intensity is most probably caused by the tensile strain-induced microscale compositional pulling and Ga segregation. And one can determine the composition variation according to the commonly known relationship between composition and band gap of Al_*x*_Ga_1−*x*_N as follows [41]:

1 $$E_{g} \left( x \right) = \left( {1 - x} \right)E_{g} \left( {{\text{GaN}}} \right) + xE_{g} \left( {{\text{AlN}}} \right) - bx\left( {1 - x} \right),$$

where $E_{g} \left( {{\text{GaN}}} \right)$ is the band gap of GaN (~ 3.5 eV), $E_{g} \left( {{\text{AlN}}} \right)$ is the band gap of AlN (~ 6.1 eV) and *b* (~ 1 eV) is the bowing parameter. The corresponding Al composition of Position A and Position C is 0.4475 and 0.4295, and the difference value is about 0.0180 according to the emission energies.

To verify our deduction, the microscale strain field distribution of a hexagonal area of MQWs was characterized by the Raman measurement. As shown in Fig. 4d, e, the Raman spectra of seven positions from the outside to the center of a hexagonal area were collected. The spectra show typical *E*_2_(GaN-like) mode around 585 cm^−1^ that allowed by Raman selection rule [42]. As has been reported, the Raman shift of *E*_2_(GaN-like) mode is sensitive to the biaxial strain condition of AlGaN [43]. And the *E*_2_(GaN-like) phonon frequency shift Δ*ω* can be used to determine the biaxial stress *σ*_*xx*_ of the AlGaN with the following relationship [44]:

2 $$\sigma_{xx} = \frac{\Delta \omega }{{4.3}}\left( {{\text{cm}}^{ - 1} \;{\text{GPa}}^{ - 1} } \right),$$

where *∆ω* represents the phonon frequency shift with respect to that in the unstressed AlGaN. According to previous work [45], the phonon frequency of unstressed AlGaN with Al composition similar to our MQWs (0.47 average Al composition) is located in 593.3 cm^−1^, then the biaxial stress of Point 1 to 7 can be evaluated according to the above relation. As can be seen from Fig. 4e, f, all the Raman frequencies of seven points are located in the lower frequency side compared with the un-strained frequency, indicating that the sample experiences a large tensile stress. Moreover, from Point 1 to Point 4 (similar position with Position C in the CL images), the Raman frequency shifted from 585.9 to 583.5 cm^−1^, and the tensile stress increased from 1.72 to 2.28 GPa. From Point 4 to Point 7 (similar position with Position A in the CL images), the Raman frequency shifted back to 585.9 cm^−1^, and tensile stress decreased to the similar value of Position 1. Raman results clearly demonstrate that the boundary area between two grains experiences a larger tensile stress compared with grain area itself. As discussed above, the additional tensile stress will further pull out Al atom from AlGaN and result in a higher Ga composition, finally leading to the redshift of emission energy.

It is worth noting that the tensile stress itself can introduce a reduction in band gap in AlGaN directly, and the relation between the band gap *E*_*g*_ and the stress follows the formula [46]:

3 $$E_{g} = E_{g} \left( 0 \right) + 3.6 \times 10^{ - 4} P - 1.76 \times 10^{ - 8} P^{2} ,$$

where *E*_*g*_(0) is the band gap without stress, *P* is the stress value of material. According to this relationship, we can calculate the stress-induced band gap difference Δ*E*_*g*_ between Point 4 and Point 7 (or Point 1) which is only about 0.2 meV. That is much smaller than the emission energy shift observed in the CL spectra (45 meV), meaning that the stress-induced band gap change is not the direct and main factor of emission energy shift, which should be the strain-induced microscale compositional pulling. On the other hand, the variation in AlGaN composition also can lead to the shift of *E*_2_(GaN-like) mode. It is difficult to distinguish the contributions from composition or stress for the Raman shift. However, we still can make a qualitative analysis based on the tendency of *E*_2_(GaN-like) along with composition [42, 45]. According to the CL emission results, the corresponding Al composition of center and edge of a hexagonal area is about 0.4475 and 0.4295, and the difference value is about 0.0180. This little variation in composition can introduce a small shift about 1.11 cm^−1^ for *E*_2_(GaN-like) mode based on previous reports [42, 45]. This value is smaller than the experimentally observed shift value (2.4 cm^−1^), indicating that the observed shift of *E*_2_(GaN-like) mode is mostly contributed from the additional tensile stress.

**Fig. 5:**
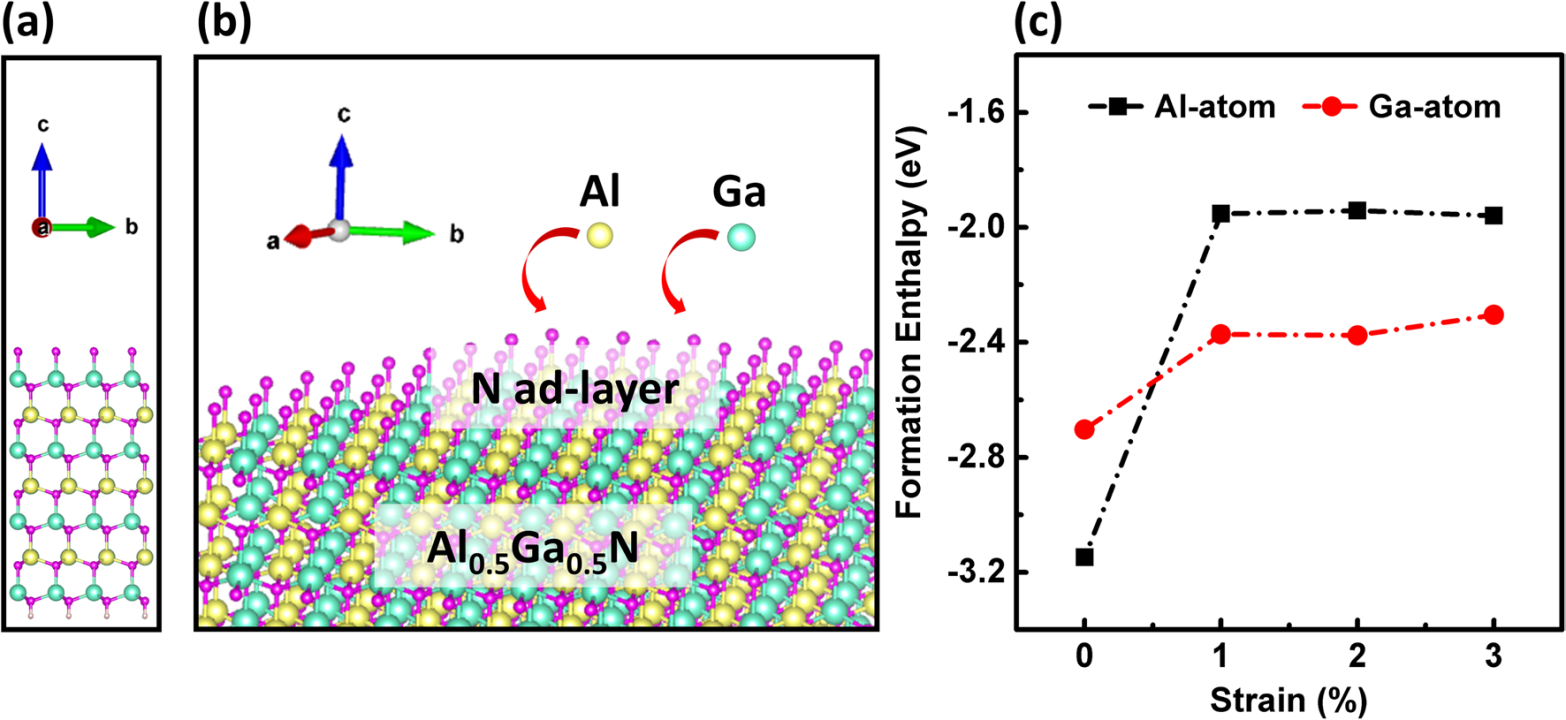
**a** Al_0.5_Ga_0.5_N slab model generated by 4*a* × 4*b* × 3*c* primitive cells for the simulation. **b** Schematic of Al_0.5_Ga_0.5_N slab model with Al atom or Ga atom adsorbed onto the surface with *N* ad-layer. **c** Formation enthalpy of Al atom and Ga atom adsorbed onto the Al_0.5_Ga_0.5_N surface as a function on the tensile strain level

In spite of a lot of works have reported the strain-induced compositional pulling effect, there still lack of a detail analysis about the inside mechanism. To further provide a clear understanding of this phenomenon, the formation enthalpies of the Al/Ga atom adsorbed onto Al_0.5_Ga_0.5_N surface under different tensile strain levels were evaluated by performing first principle total-energy calculation, using the following formula [47]:

4 $$E_{f} = \left( {E_{{{\text{tot}}}} - E_{{{\text{clean}}}} } \right) - \Delta n_{i} \mu_{i} = \left( {E_{{{\text{tot}}}} - E_{{{\text{clean}}}} } \right) - \Delta n_{{{\text{Ga}}}} \mu_{{{\text{Ga}}}} - \Delta n_{{{\text{Al}}}} \mu_{{{\text{Al}}}} ,$$

where *E*_tot_ and *E*_clean_ represent the total energies of the absorbed and clean Al_0.5_Ga_0.5_N surface, $\Delta {n}_{i}$ denotes the difference between the number of atoms in the absorbed and clean Al_0.5_Ga_0.5_N surface, and ${\mu }_{i}$ is the chemical potentials of Ga and Al atom, respectively. Figure 5a–c shows the simulation model and results. Under the un-strained condition, the formation enthalpy of adding Al atom is smaller about 0.44 eV than adding Ga atom on the top of *N* ad-layer. This behavior indicates that Al atom is much easier to be incorporated into the lattice, which is agreed with the nature of low surface migration of Al atom [48]. When the tensile strain is applied into the AlGaN slab, the formation enthalpies of both Al and Ga atoms increase greatly due to the introduction of strain energy. Most importantly, the enthalpy increment of Al atom (1.19 eV at 1% strain) is much larger than Ga atom (0.33 eV at 1% strain), which leads to a higher formation enthalpy about 0.42 eV of Al atom than Ga atom. These results confirm that under a tensile strain, the incorporation of Ga atom into the lattice is more thermodynamically stable and favorable than Al atom. Therefore, the mechanism of strain-induced microscale compositional pulling effect and the origin of lower Al composition, i.e., the Ga segregation, in MQWs are theoretically explained.

**Fig. 6:**
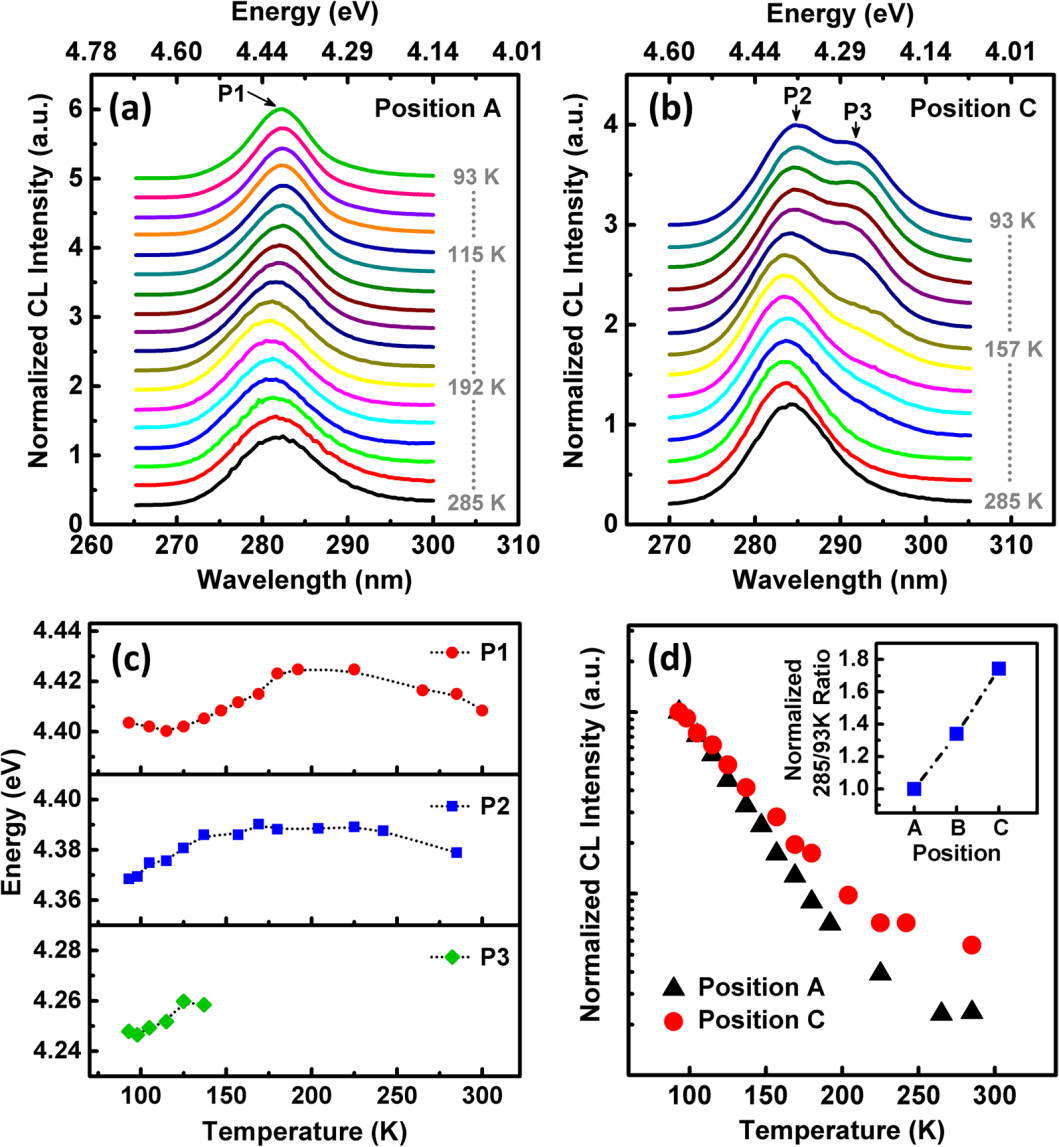
Normalized temperature-dependent CL spectra taken in the **a** Position A and **b** Position C. **c** Variation in P1, P2 and P3 energy with temperature, which were extracted by Gaussian fitting. **d** Temperature dependence of the integrated CL intensity of Position A and C. The inset shows the normalized 285/93 K CL integral intensity ratio of three positions

It is important to explore the role of strain-induced microscale compositional pulling on the optical properties that have great influence on the quantum efficiency of MQWs. Thus, we further measured the temperature-dependent CL spectra of Position A and C in a range of 285 K to 93 K, as shown in Fig. 6a, b, respectively. In order to compare the peak positions and shape, all CL spectra were normalized. In Position A, there is only one peak, which can be attributed to P1, i.e., the intrinsic emission of MQWs. In Position C, there is also only one peak at high temperature and can be attributed to P2, i.e., the emission originated from compositional pulling and Ga segregation. Interestingly, with temperature lower than 157 K, another new peak appears in the longer wavelength side of P2. For convenience, we denote this new peak as P3. Figure 6c shows the variation in P1, P2 and P3 peak energy as a function of temperature, which were extracted from spectra by Gaussian fitting.

As the temperature increases, P1 exhibits a redshift from 4.404 eV at 93 K to 4.400 eV at 115 K firstly, then blue shifts to 4.425 eV at 192 K, finally redshifts again to 4.415 eV at 285 K, shows a typical “S-shape” temperature-dependent behavior. For P2, as the temperature increases from 93 to 225 K, the energy blue shifts from 4.368 to 4.389 eV, then redshifts to 4.379 eV at 285 K. The “S-shape” behavior is commonly observed in the InGaN epilayer [49], InGaN-based MQWs [50], and AlGaN epilayer [51]. In general, this phenomenon is explained by the carrier recombination dynamic with localization effect [50]. For the first redshift process at low temperature, the radiative recombination process is dominant and the carrier lifetime increases with temperature, hence the carriers have more opportunity to relax down into the lower energy tail states, leading to the redshift with increasing temperature. For the blueshift process at higher temperature, the carrier lifetimes decrease greatly and the dissociation rate increases, so the carriers recombine quickly before reaching the lower energy tail states, resulting in the observed blueshift. Further increasing temperature to room temperature, the nonradiative recombination becomes dominant process and the carrier lifetimes decrease to a constant, then the blueshift behavior is reduced. Besides, due to the thermal disturbance-induced delocalization, the localization carriers become the free carriers, which enhances the temperature-induced band gap shrinkage, finally the emission energy redshift again with increasing temperature. This analysis shows that the second inflection point of temperature-dependent emission energy represents the delocalization of most carriers. For P2, the temperature of this inflection point between blueshift and final redshift is higher than P1, which demonstrates that the stronger carrier localization centers exist around the Position C. As for the P3 at low temperature, at present, we are still not quite sure about its exact origin, but it may come from the emission of bound exciton around the area of grain boundary [52].

The PL or CL intensity ratio between the low and high temperature is closely related to the internal quantum efficiency (IQE) and often used to estimate the efficiency. Figure 6d shows the temperature dependence of the integrated CL intensity of Position A and C, and the intensity is normalized with that at the lowest temperature. In the whole range of temperature, Position C exhibits a higher intensity ratio than Position A. The normalized 285/93 K ratio of Position C is about 1.7 times higher compared with Position A, indicating that Position C has a higher quantum efficiency than Position A. This is because that the strain-induced local Ga segregation can provide stronger three-dimensional carrier localization to enhance the radiation recombination rate and suppress the outflow of carriers toward nonradiative centers, finally boost the overall luminescence efficiency of MQWs [53, 54]. It has been reported that using the misoriented sapphire substrate can promote the formation of step bunches in the AlGaN MQWs, thus introduce Ga composition inhomogeneity and locally varied potential minima, finally enhance the DUV luminescence [55]. Enlighted by these works, we believe that it is also possible to further increase the proportion of the high efficiency P2 emission by introducing more step bunches and grain coalescence boundaries via using misoriented sapphire substrate.

## Conclusions

In summary, a systematic study that combined characterization and simulation was carried out for the strain-induced microscale compositional pulling effect on the structural and optical properties of high Al content AlGaN MQWs. The CL spectrum of the MQWs demonstrates a typical asymmetrical feature. Microscopic CL mapping shows that the asymmetrical emission is caused by two different type of emissions, which have inhomogeneous spatial distributions and diverse origins. Microscopic Raman spectra reveal that a large tensile strain is introduced during the epitaxial growth of AlGaN MQWs, due to the grain boundary formation, coalescence and growth. The presence of this tensile strain results in the microscale inhomogeneous compositional pulling and Ga segregation, which is further confirmed by the lower formation enthalpy of Ga atom than Al atom on AlGaN slab using first principle simulations. This strain-induced microscale compositional pulling leads to the asymmetrical feature of emission spectra and a local variation in the emission energy and intensity of AlGaN MQWs. Moreover, the area of Ga segregation shows a higher emission efficiency compared with the intrinsic area of MQWs due to stronger three-dimensional carrier localization, which is benefit for fabricating high-performance AlGaN-based DUV LEDs.

## Data Availability

The data and the analysis in the current work are available from the corresponding authors on reasonable request.

## Abbreviations

MQWs: Multiple quantum wells
DUV: Deep-ultraviolet
LED: Light-emitting diode
EQE: External quantum efficiency
MOVPE: Metal organic vapor phase epitaxy
AFM: Atomic force microscope
XRD: X-ray diffraction
TEM: Transmission electron microscope
CL: Cathodoluminescence
DFT: Density-functional theory
TMA: Trimethylaluminum
TMG: Trimethylgallium
NH_3_: Ammonia
SiH_4_: Silane
H_2_: Hydrogen
PALE: Pulsed atomic layer epitaxy method
SL: Superlattice
VASP: Vienna ab-initio simulation package
PBE: Perdew–Burke–Ernzerhof
GGA: Generalized gradient approximation
RMS: Root-mean-square
IQE: Internal quantum efficiency

## References

[1] Li D, Jiang K, Sun X, Guo C (2018). AlGaN photonics: recent advances in materials and ultraviolet devices. Adv Opt Photon.

[2] Kneissl M, Seong T-Y, Han J, Amano H (2019). The emergence and prospects of deep-ultraviolet light-emitting diode technologies. Nat Photon.

[3] Khan A, Balakrishnan K, Katona T (2008). Ultraviolet light-emitting diodes based on group three nitrides. Nat Photon.

[4] Liu S, Luo W, Li D (2021). Sec-eliminating the SARS-CoV-2 by AlGaN based high power deep ultraviolet light source. Adv Funct Mater.

[5] Lee EC, Wada NI, Grabowski MK (2020). The engines of SARS-CoV-2 spread. Science.

[6] Mackey TK, Contreras JT, Liang BA (2014). The Minamata convention on mercury: attempting to address the global controversy of dental amalgam use and mercury waste disposal. Sci Total Environ.

[7] Li J, Gao N, Cai D (2021). Multiple fields manipulation on nitride material structures in ultraviolet light-emitting diodes. Light Sci Appl.

[8] Wang CH, Ke CC, Lee CY (2010). Hole injection and efficiency droop improvement in InGaN/GaN light-emitting diodes by band-engineered electron blocking layer. Appl Phys Lett.

[9] Zhang Z-H, Huang Chen S-W, Chu C (2018). Nearly efficiency-droop-free AlGaN-based ultraviolet light-emitting diodes with a specifically designed superlattice p-type electron blocking layer for high Mg doping efficiency. Nanoscale Res Lett.

[10] Li L, Zhang Y, Xu S (2017). On the hole injection for III-nitride based deep ultraviolet light-emitting diodes. Materials (Basel).

[11] Shatalov M, Sun W, Lunev A (2012). AlGaN deep-ultraviolet light-emitting diodes with external quantum efficiency above 10%. Appl Phys Express.

[12] Takano T, Mino T, Sakai J (2017). Deep-ultraviolet light-emitting diodes with external quantum efficiency higher than 20% at 275 nm achieved by improving light-extraction efficiency. Appl Phys Express.

[13] Wang T-Y, Tasi C-T, Lin C-F, Wuu D-S (2017). 85% internal quantum efficiency of 280-nm AlGaN multiple quantum wells by defect engineering. Sci Rep.

[14] Dong P, Yan J, Wang J (2013). 282-nm AlGaN-based deep ultraviolet light-emitting diodes with improved performance on nano-patterned sapphire substrates. Appl Phys Lett.

[15] Hirayama H, Norimatsu J, Noguchi N (2009). Milliwatt power 270 nm-band AlGaN deep-UV LEDs fabricated on ELO-AlN templates. Phys Status Solidi.

[16] Jinschek JR, Erni R, Gardner NF (2006). Local indium segregation and bang gap variations in high efficiency green light emitting InGaN/GaN diodes. Solid State Commun.

[17] Lai Y-L, Liu C-P, Lin Y-H (2006). Origins of efficient green light emission in phase-separated InGaN quantum wells. Nanotechnology.

[18] Cho HK, Lee JY, Song JH (2002). Influence of strain-induced indium clustering on characteristics of InGaN/GaN multiple quantum wells with high indium composition. J Appl Phys.

[19] de Sousa Pereira SM, O’Donnell KP, da Costa Alves EJ (2007). Role of nanoscale strain inhomogeneity on the light emission from InGaN epilayers. Adv Funct Mater.

[20] Jain SC, Willander M, Narayan J, Van OR (2000). III–Nitrides: growth, characterization, and properties. J Appl Phys.

[21] Stringfellow GB (1972). The importance of lattice mismatch in the growth of Ga x In 1–x P epitaxial crystals. J Appl Phys.

[22] Pereira S, Correia MR, Pereira E (2001). Compositional pulling effects in InxGa1-xN/GaN layer: a combined depth-resolved cathodoluminescence and Rutherford backscattering/channeling study. Phys Rev B.

[23] He C, Qin Z, Xu F (2016). Mechanism of stress-driven composition evolution during hetero-epitaxy in a ternary AlGaN system. Sci Rep.

[24] Tsai Y-L, Wang C-L, Lin P-H (2003). Observation of compositional pulling phenomenon in AlxGa1−xN (0.4 < x < 1.0) films grown on (0001) sapphire substrates. Appl Phys Lett.

[25] Liu B, Zhang R, Zheng JG (2011). Composition pulling effect and strain relief mechanism in AlGaN/AlN distributed Bragg reflectors. Appl Phys Lett.

[26] Gonze X, Beuken J-M, Caracas R (2002). First-principles computation of material properties: the ABINIT software project. Comput Mater Sci.

[27] Segall MD, Lindan PJD, Probert MJ (2002). First-principles simulation: ideas, illustrations and the CASTEP code. J Phys Condens Matter.

[28] Perdew JP, Burke K, Ernzerhof M (1996). Generalized gradient approximation made simple. Phys Rev Lett.

[29] Moram MA, Vickers ME (2009). X-ray diffraction of III-nitrides. Rep Prog Phys.

[30] Akasaki I, Amano H, Koide Y (1989). Effects of ain buffer layer on crystallographic structure and on electrical and optical properties of GaN and Ga1−xAlxN (0 < x ≦ 0.4) films grown on sapphire substrate by MOVPE. J Cryst Growth.

[31] Hiramatsu K, Itoh S, Amano H (1991). Growth mechanism of GaN grown on sapphire with A1N buffer layer by MOVPE. J Cryst Growth.

[32] Hiramatsu K, Kawaguchi Y, Shimizu M (1997). The composition pulling effect in MOVPE grown InGaN on GaN and AlGaN and its TEM characterization. MRS Internet J Nitride Semicond Res.

[33] Ponce FA (1997). Defects and Interfaces in GaN Epitaxy. MRS Bull.

[34] Chang L, Lai SK, Chen FR, Kai JJ (2001). Observations of Al segregation around dislocations in AlGaN. Appl Phys Lett.

[35] Chang L, Lai SK, Chen FR, Kai JJ (2001). Observations of segregation of Al in AlGaN alloys. Phys Status Solidi.

[36] Massabuau FCP, Rhode SL, Horton MK (2017). Dislocations in AlGaN: core structure, atom segregation, and optical properties. Nano Lett.

[37] Liu W, Carlin J-F, Grandjean N (2016). Exciton dynamics at a single dislocation in GaN probed by picosecond time-resolved cathodoluminescence. Appl Phys Lett.

[38] He C, Qin Z, Xu F (2016). Effect of stress on the Al composition evolution in AlGaN grown using metal organic vapor phase epitaxy. Appl Phys Express.

[39] Sheldon B, Bhandari A, Bower A (2007). Steady-state tensile stresses during the growth of polycrystalline films. Acta Mater.

[40] Floro JA, Chason E, Cammarata RC, Srolovitz DJ (2002). Physical origins of intrinsic stresses in Volmer–Weber thin films. MRS Bull.

[41] Nepal N, Li J, Nakarmi ML (2005). Temperature and compositional dependence of the energy band gap of AlGaN alloys. Appl Phys Lett.

[42] Davydov VY, Goncharuk IN, Smirnov AN (2002). Composition dependence of optical phonon energies and Raman line broadening in hexagonal AlxGa1-xN alloys. Phys Rev B.

[43] Harima H (2002). Properties of GaN and related compounds studied by means of Raman scattering. J Phys Condens Matter.

[44] Zhang L, Yu J, Hao X (2015). Influence of stress in GaN crystals grown by HVPE on MOCVD-GaN/6H-SiC substrate. Sci Rep.

[45] Zheng J, Li J, Zhong Z (2017). Effect of electrical injection-induced stress on interband transitions in high Al content AlGaN MQWs. RSC Adv.

[46] Van Camp PE, Van Doren VE, Devreese JT (1991). High-pressure properties of wurtzite- and rocksalt-type aluminum nitride. Phys Rev B.

[47] Zhuang Q, Lin W, Yang W (2013). Defect suppression in AlN epilayer using hierarchical growth units. J Phys Chem C.

[48] Lobanova AV, Yakovlev EV, Talalaev R (2008). Growth conditions and surface morphology of AlN MOVPE. J Cryst Growth.

[49] Moon Y-T, Kim D-J, Park J-S (2001). Temperature dependence of photoluminescence of InGaN films containing In-rich quantum dots. Appl Phys Lett.

[50] Cho Y-H, Gainer GH, Fischer AJ (1998). “S-shaped” temperature-dependent emission shift and carrier dynamics in InGaN/GaN multiple quantum wells. Appl Phys Lett.

[51] Kazlauskas K, Žukauskas A, Tamulaitis G (2005). Exciton hopping and nonradiative decay in AlGaN epilayers. Appl Phys Lett.

[52] He C, Qin Z, Xu F (2015). Free and bound excitonic effects in Al0.5Ga0.5N/Al0.35Ga0.65N MQWs with different Si-doping levels in the well layers. Sci Rep.

[53] Kojima K, Nagasawa Y, Hirano A (2019). Carrier localization structure combined with current micropaths in AlGaN quantum wells grown on an AlN template with macrosteps. Appl Phys Lett.

[54] Hayakawa M, Ichikawa S, Funato M, Kawakami Y (2019). AlxGa1−xN-based quantum wells fabricated on macrosteps effectively suppressing nonradiative recombination. Adv Opt Mater.

[55] Sun H, Mitra S, Subedi RC (2019). Unambiguously enhanced ultraviolet luminescence of AlGaN wavy quantum well structures grown on large misoriented sapphire substrate. Adv Funct Mater.

